# STE20/PAKA Protein Kinase Gene Releases an Autoinhibitory Domain through Pre-mRNA Alternative Splicing in the Dermatophyte *Trichophyton rubrum*

**DOI:** 10.3390/ijms19113654

**Published:** 2018-11-20

**Authors:** Eriston V. Gomes, Julio C. Bortolossi, Pablo R. Sanches, Niege S. Mendes, Nilce M. Martinez-Rossi, Antonio Rossi

**Affiliations:** 1 Department of Genetics, Ribeirão Preto Medical School, São Paulo University, Ribeirão Preto, São Paulo 14049-900, Brazil; eristongomes@hotmail.com (E.V.G.); bortolossijc@usp.br (J.C.B.); psanches@usp.br (P.R.S.); niege.mendes@hotmail.com (N.S.M.); anrossi@usp.br (A.R.); 2 Department of Biofunctional, Center of Higher Education Morgana Potrich Eireli, Morgana Potrich College, Mineiros, Goiás 75830-000, Brazil

**Keywords:** *Trichophyton rubrum*, signaling pathway, MAPK, STE/PAK kinase, alternative splicing, intron retention, enzyme activation, undecanoic acid

## Abstract

Signaling pathways are highly diverse in filamentous fungi, allowing the cells to receive and process ambient information. Interaction of components from different pathways results in signaling networks. The mitogen-activated protein kinase (MAPK) pathway is dependent on phosphorylation that is accomplished by kinase proteins. Thus, the STE/PAK protein kinase family plays essential roles in MAPK signal transduction, regulating several cellular functions. The STE/PAK protein displays an autoinhibitory (Cdc42/Rac interactive binding—CRIB) domain on its N-terminal portion, which interacts with the C-terminal catalytic kinase domain. Based on current knowledge, for the STE/PAK kinase to be activated, molecular signals (e.g., interaction with the activated form of Rac1 and Cdc42 proteins) or proteolytic cleavage by caspase 3 is necessary. Both mechanisms release the kinase domain from the CRIB interaction. Here, we hypothesize a novel molecular mechanism for the activation of STE20/PAKA kinase in *Trichophyton rubrum* based on an alternative pre-mRNA splicing process. Our data suggest that, because of the retention of intron 1 of this gene, it is theoretically possible that the translation of STE20/PAKA kinase will be free of its autoinhibitory CRIB domain. These findings indicate a rapid response system to environmental changes. Furthermore, STE20/PAKA may be a potential *T. rubrum* virulence factor and an interesting target for new drugs against dermatophytes.

## 1. Introduction

Regardless of lifestyle, living organisms including filamentous fungi monitor various environmental signals. It is imperative to sense a broad range of stimuli, including the nutritional availability status and the presence of hosts or predators. Thus, when facing different kinds of signals (e.g., light, temperature, pheromones, and chemical compounds), organisms respond adequately to environmental changes [1,2]. Kinase proteins play a fundamental role in the transduction of cellular signals, from the receptors to the active response, which may include transcription and translation of effector proteins [3]. Mitogen-activated protein kinase (MAPK) pathways are directly or indirectly involved in the molecular responses to these stimuli. These processes display an evolutionarily conserved mechanism from yeast to humans, acting as a switch, turning on or off the activity of its substrate proteins [4]. Pathogenic fungi carrying defective mutations in genes that integrate the MAPK pathway show reduced virulence in animal models of infection [5]. The p21 activated kinase (PAK), a member of the Ste20-related kinase family, was initially isolated from rat brain. The PAK protein displays a conserved N-terminal domain, which serves as a binding site for the activated form of Rac1 and Cdc42 proteins (Cdc42/Rac interactive binding—CRIB), acting as an autoinhibitory mechanism for the C-terminal kinase domain [6]. In the MAPK pathway, Ste20p usually phosphorylates Ste11p (a MAPKKK protein) which follows the MAPK cascade and regulates several cellular responses, such as mating, cell wall maintenance, osmotic regulation, filamentous growth, and virulence [7,8]. Under apoptotic stress, there is an interesting transient and constitutive activation of mammalian PAK2 by caspase 3 (CPP32) cleavage, leading to the transcriptional inhibition of several growth-related genes [9,10,11].

Intron retention (IR) in filamentous fungi is the most common alternative pre-mRNA splicing mechanism (AS) and may be modulated in response to nutrient signaling, as described for the MAPK protein coded by mak-2 gene in *Neurospora crassa* [12]. As a dermatophyte, *Trichophyton rubrum* needs to probe the surrounding environment, obtain its nutrients, and consequently develop hyphae with adequate structures to penetrate the host tissue. Moreover, several stress factors must be considered, such as temperature, lack of moisture, ultraviolet light, and the host defense system. Therefore, the MAPK signaling pathway is of fundamental importance for the development and successful interaction and tissue invasion by the fungus [13]. Here, we describe the retention of intron 1 during transcription of the *pakA* gene and propose a molecular mechanism for the activation of the *T. rubrum* PAKA kinase as an alternative to the mammalian caspase 3 cleavage through AS. We suggest that retention of intron 1 generates, based on the putative fungal STE20/PAK protein activation and RNA-Seq transcriptome analysis, both a premature stop codon in its respective mRNA and a new start codon soon after that, nonetheless preserving most of the original protein amino acid sequence. Subsequently, at least two independent polypeptides are theoretically produced. The first one contains the regulatory CRIB domain, and the second one—displaying the entire kinase catalytic domain—is free of the regulatory domain. This observation suggests that, depending on the external stimulus, the MAPK metabolic pathway may be activated with variable intensities and regulated by a different set of molecules, such as the production of a PAKA kinase independent of the Cdc42/Rac system regulation. Intron 1 retention is also modulated by the presence of the antifungal agent undecanoic acid (UDA), probably for a rapid response to environmental changes or stress conditions, which also add support to our hypothesis. These findings may help to clarify the mechanism of adaptation and host-fungi interactions, indicating specific novel targets for the development of more efficient drugs in the fight against dermatophytoses.

## 2. Results

### 2.1. T. rubrum pakA/Ste20 Kinase Gene

**Figure 1:**
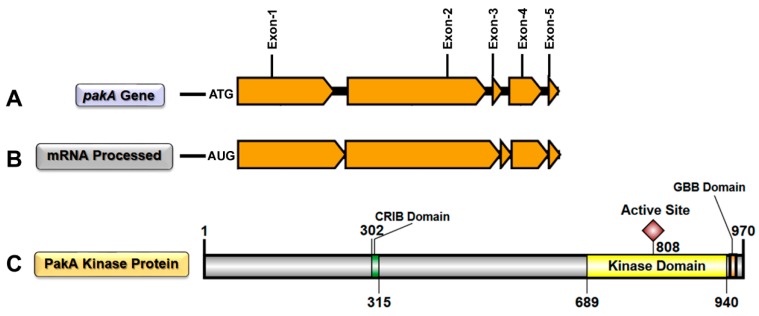
Graphical representation of complete *pakA*/*Ste20* gene transcription/splicing/translation in *T. rubrum*. (**A**) Graphical representation of the *pakA*/*Ste20* kinase gene with its five exons (dark yellow) and four introns (black); (**B**) Graphical representation of mRNA transcribed/spliced from the *pakA*/*Ste20* kinase gene; (**C**) Graphical representation of functional STE20/PAKA protein, with its respective domains, translated from processed mRNA.

*T. rubrum pakA*/*Ste20* (TERG_03042) is a 3239-bp gene. The complete splicing process of *pakA* transcription generates a mRNA with 2912 bases, which encodes a 970-amino acid protein. According to ScanProsite, a web-based tool for detecting signature matches in protein sequences [14,15], PAKA exhibits the CRIB regulatory domain on its N-terminal portion, between amino acids 302 and 315. Furthermore, both the catalytic site and the G*β* binding (GBB) domains are located at the C-terminal region (Figure 1). PAKA also presents other intriguing areas, such as six regions rich in specific amino acids, which may be necessary for interactions with other proteins, metabolites, small molecules, and ions (Figure S1A). Moreover, PAKA displays several putative post-translational modification sites. There are 39 phosphorylation sites: 16 sites for protein kinase C (PKC), five for cAMP/cGMP-dependent protein kinase (CAMP), and 18 for casein kinase II (CK2) (Figure S1B). There are also 27 sites for N-myristoylation (Myr), six sites for N-glycosylation (N-gly; only in N-terminal region), and one site for amidation (AMD) inside the kinase domain (Figure S1C,D). We also used EMBOSS epestfind (http://emboss.bioinformatics.nl/cgi-bin/emboss/epestfind) (accessed on 19 June 2018) another web-based tool for detecting PEST motifs as potential proteolytic cleavage sites. The results showed two potential PEST motifs: one with 15 amino acids between position 157 and 173 (PEST score: 15.52), and another with 44 amino acids between position 467 and 512 (PEST score: 8.06) (Figure S1E).

### 2.2. Transcription Retention of Introns of the pakA Kinase Gene of T. rubrum

**Figure 2:**
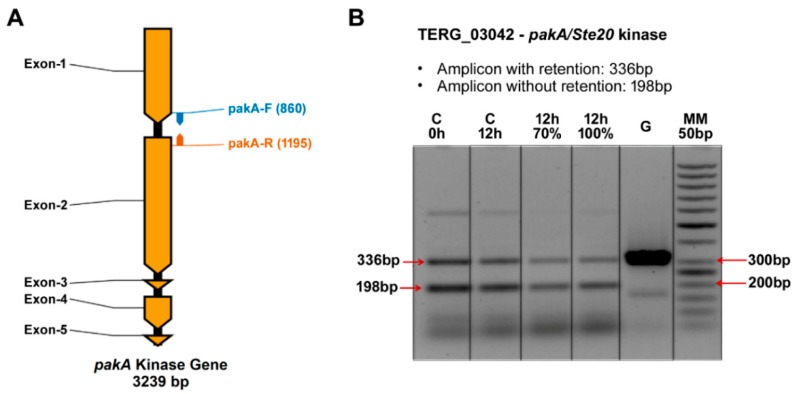
Intron retention analysis of the *T. rubrum pakA*/*Ste20* kinase gene. (**A**) Graphical representation of the *pakA*/*Ste20* kinase gene indicating its size (base pairs), five exons (dark yellow), four introns (black), and the primer-binding sites used for amplification of the region flanking intron 1 (blue and orange arrows); (**B**) Agarose gel electrophoresis (1.5%) run with qRT-PCR products from the *pakA*/*Ste20* intron 1 region. **C**: culture without the presence of UDA; **G**: amplification from genomic DNA (gene amplification positive control); **0 h**: amplification product from cDNA obtained from culture in medium lacking the drug; **70%**: amplification product from cDNA obtained from culture in medium containing 70% of the UDA MIC; **100%**: amplification product from cDNA obtained from culture in medium containing 100% of the UDA MIC; **MM**: molecular weight marker (50 bp).

Using a high-throughput RNA-sequencing (RNA-Seq) approach, we identified IR events in the transcriptional processing of the *T. rubrum pakA* gene after treatment with sub-inhibitory doses of UDA. These data indicated that 56 reads exhibited retention of intron 1 in the *pakA* transcriptional products after 12 h of UDA treatment (RNA-Seq data). To validate these data, a set of primers flanking the intron 1 region of this gene was designed and used for reverse transcription polymerase chain reaction (qRT-PCR) (Figure 2A), followed by 1.5% agarose gel electrophoresis. The amplicon generated from the genomic DNA presented a fragment of 336 bp as well as the predicted fragment that exhibits IR. The expected amplicon for the processed transcript was a 198-bp DNA fragment. The results showed that IR was present in both the control conditions (96 h pre-culture [control − 0 h] and 12 h of culture in the absence of UDA [control − 12 h]) as well in 12-h cultures with 70% and 100% of the UDA minimum inhibitory concentration (MIC) (Figure 2B). These results indicate that retention of intron 1 from *pakA* gene is a process that occurs naturally in the assayed conditions. These results were confirmed in biological triplicates (Figure S2).

**Figure 3:**
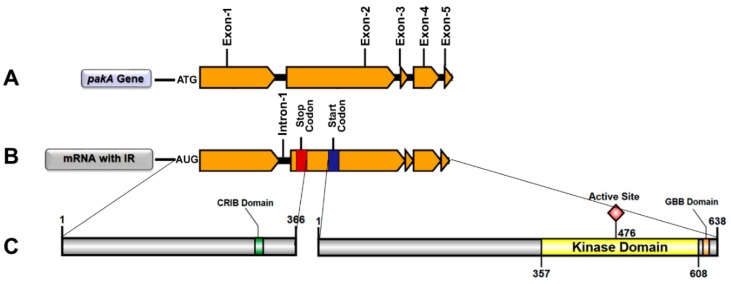
Graphical representation of alternative *pakA*/*Ste20* gene transcription/splicing/translation in *T. rubrum*. (**A**) Graphical representation of the *pakA*/*Ste20* kinase gene with its five exons (dark yellow) and four introns (black); (**B**) Graphical representation of mRNA transcribed from the *pakA*/*Ste20* kinase gene with retention of intron 1; (**C**) Graphical representation of both probable functional STE20/PAKA proteins, with their respective domains, translated from intron-retained mRNA.

In silico analysis indicated that the retention of intron 1, present in the alternative *pakA* gene transcription, generates a premature stop codon (UAA) 1098 bases downstream of the start codon. This first mRNA sequence encodes a polypeptide with 336 amino acid residues, which differs from the N-terminal portion of the native PAKA protein only in the last 42 amino acids. Interestingly, 33 bases downstream from the first stop codon defines a new start codon followed by a sequence which encodes a protein with 638 amino acid residues. The alignment of this amino acid sequence indicated that it is identical to the C-terminal portion of the native PAKA protein, with both the catalytic site and the GBB domains (Figure 3) as well as the translational modification sites but without the regulatory CRIB domain.

### 2.3. UDA Affects the Transcription Process of the pakA/Ste20 Gene in T. rubrum

**Figure 4:**
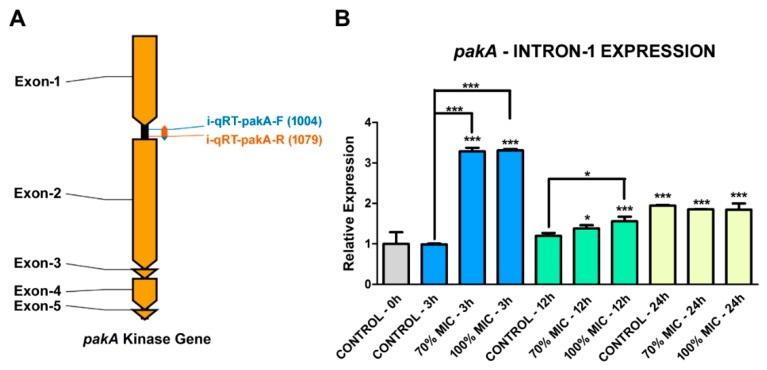
qRT-PCR analysis of the intron 1 retention levels of the *T. rubrum pakA/ste20* gene. (**A**) Graphical representation of the *pakA/Ste20* kinase gene with its five exons (dark yellow), four introns (black), and the primer-binding sites used for amplification of the intron 1 retention (blue and orange arrows). (**B**) Intron 1 retention levels of the *T. rubrum pakA/ste20* gene, incubated in the presence of 70% and 100% UDA MIC for 3, 12, and 24 h using ANOVA followed by Tukey’s *post hoc* test (* *p* < 0.05; *** *p* < 0.001).

A set of quantitative RT-PCR (qRT-PCR) primers was designed to target within the sequence of intron 1 of the *pakA* gene to determine the influence of UDA in its retention (Figure 4A). The results showed no significant variation in retention of intron 1 during the first 12 h in the absence of UDA, considering the control − 0 h (just after the 96-h pre-cultivation, without UDA) as the reference condition. However, an increase in retention was observed after 24 h of cultivation in the same situation (Figure 4B). Conversely, there was a strong retention peak after 3 h of culture in the presence of the drug regardless of its concentration (Figure 4B). These results indicated that UDA modulates intron 1 retention in the *pakA* kinase gene of *T. rubrum*. Also, after 12 h of culturing in the presence of UDA, the retention levels remained statistically representative, maintaining the IR higher than in the control conditions, especially with 100% MIC (Figure 4B). After 24 h of cultivation, there was a relative stabilization in IR levels considering both culture situations (with and without UDA), but with higher IR levels in culture with UDA when compared with that of the control condition (control − 0 h) (Figure 4B).

**Figure 5:**
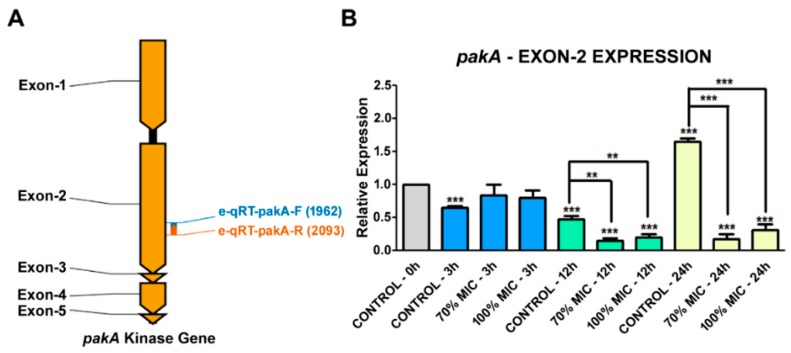
qRT-PCR analysis of the *T. rubrum pakA*/*ste20* gene expression levels. (**A**) Graphical representation of the *pakA*/*Ste20* kinase gene with its five exons (dark yellow), four introns (black), and the primer-binding sites used for gene expression analysis (blue and orange arrows). (**B**) Expression levels of the *T. rubrum pakA*/*ste20* gene, incubated in the presence of 70% and 100% UDA MIC for 3, 12, and 24 h using ANOVA followed by Tukey’s *post hoc* test (** *p* < 0.01; *** *p* < 0.001).

We also analyzed the relative expression levels of the *pakA*/*Ste20* kinase gene in *T. rubrum* under different culture conditions, using a set of qRT-PCR primers designed within the exon 2 sequence of this gene (Figure 5A). Interestingly, there was a decrease in *pakA* expression after culturing the fungus for 12 h, being more pronounced in the presence of UDA, suggesting that the drug forced a stronger down-regulation. This same UDA effect was observed after 24 h (Figure 5B). These results indicated that UDA affects the MAPK pathway somewhere up-stream of the STE20/PAKA signaling cascade or perhaps the transcription of some transcription factor involved in this pathway.

## 3. Discussion

MAPK is a component of an evolutionarily well-conserved signaling pathway, generally involved in a range of cellular process [16]. Different stimuli may activate its signaling cascade, leading to several cellular responses, such as cytoskeletal architecture, cytokinesis, motility, cell-cycle progression, hyphal growth, differentiation of asexual development structures, stress response, sterol homeostasis, and virulence [8,17,18]. In this context, STE/PAK kinase has a fundamental role. In yeast, genetic evidence suggests that Ste20p functions as a G-protein (*βγ* subunit) effector of downstream signaling components, such as Ste11p [19,20]. Thus, it is possible that this signaling cascade model can be extrapolated to the *T. rubrum* MAPK pathway because in silico data indicated high identity of STE20/PAKA kinase with its *Saccharomyces cerevisiae* orthologue (72%). We also observed the presence of the GBB domain in the *T. rubrum* STE20/PAKA C-terminal region (Figure 1C). This specific domain was described as a linker of Ste20p to the G*β* subunit of the *S. cerevisiae* heterotrimeric G-protein [21]. It seems that STE20/PAKA is quite a promiscuous molecule since it displays regions rich in specific amino acids (Figure S1A) which enable its interaction with a range of other regulatory proteins and with molecules with different functions. This type of interaction has been described for the kinase signaling pathway activation. For instance, the serine-rich domain of the interleukin-2 receptor β chain is crucial for the activation of both tyrosine kinase and phosphatidylinositol-3-kinase in the transduction of the human IL-2 cellular proliferation signal [22,23,24]. Moreover, *T. rubrum* STE20/PAKA showed several sites for post-translational modifications (PTMs), such as phosphorylation, Myr, N-gly, and AMD (Figure S1B–D). These PTMs may allow a wide range of possible three-dimensional configurations and an excellent mechanism for the regulation of catalytic activity of the protein, being able to perform several biological functions in response to specific physiological requirements [25,26,27]. For instance, proper protein glycosylation was necessary to promote MAPK signal fidelity in *S. cerevisiae* [16]. Myr has been associated with protein kinase activation/deactivation, membrane interaction, or localization of the enzyme near membrane-bound substrates [28,29,30,31]. AMD may modify the physicochemical properties of the bioactive peptide, being essential for receptor recognition, signal transduction, and other biological functions [32,33,34]. Among the PTMs, phosphorylation is the most intriguing, considering that the activation of most kinase proteins depends on the autophosphorylation of their activation loop. This modification induces the conversion from an inactive to active conformation, and it still occurs in the protein’s inactive state [35]. As reported in *S. cerevisiae*, the binding of Cdc42p to the Ste20p CRIB site releases the kinase domain, activating its autophosphorylation process and consequently its catalytic activation [8]. Conversely, mammalian PAK2 activation occurs by the caspase 3 cleavage mechanism. This process generates two PAK2 (p27 and p34) fragments, with the entire kinase catalytic domain in the p34 fragment, free from the inhibitory CRIB domain. Consequently, this enzyme becomes active after its autophosphorylation [11,12,13,14,15,16,17,18,19,20,21,22,23,24,25,26,27,28,29,30,31,32,33,34,35,36].

IR is the leading AS mechanism in fungi, with critical roles in different fungal aspects, such as development, reproduction, mitochondrial functions, and signal transduction. However, its contribution to fungal biology still requires further studies [37,38,39,40]. Recently, using the same RNA-Seq data of the present work, our group published AS events in a *T. rubrum* strain. An IR event was presented in the gene encoding the enzyme inosine monophosphate dehydrogenase (IMPDH; TERG_06846), which is involved in purine biosynthesis, as confirmed by qRT-PCR [41]. However, in this case, and in most circumstances described in fungi, the IR process generated an altered reading frame code and, consequently, smaller proteins with disrupted functional domains. Thus, a premature stop codon may activate the nonsense-mediated decay (NMD) pathway, thereby resulting in mRNA degradation before its translation or the formation of truncated proteins [12].

Conversely, functional alternative splicing has been described in fungi. For instance, two functional serine/threonine phosphatase (PTC7) isoforms can be produced by IR in *S. cerevisiae* strains. In this model, both proteins showed distinct functions, working in different cellular compartments, specifically in mitochondria and the nuclear envelope for the spliced and unspliced mRNA, respectively [42].

Here, we hypothesize a novel mechanism for the activation of *T. rubrum* STE20/PAKA through alternative pre-mRNA splicing. We considered that, because of alternative splicing, the translation of STE20/PAKA free from its autoinhibitory CRIB domain is possible (Figure 3). This hypothesis is supported by the RNA-Seq data that presented isoform transcripts with intron 1 retained, validated through qRT-PCR (Figure 2). Furthermore, although the *T. rubrum* Ste20/pakA kinase alternative transcript displays a premature stop codon, the subsequent reading frame is conserved. Thus, it is plausible that this mRNA may be preserved from degradation and subsequently translated, producing two independent proteins/polypeptides, because bicistronic and polycistronic transcripts have already been described in fungi [43,44]. Our data also suggested that IR by *T. rubrum Ste20*/*pakA* is a natural process, considering the transcription of both isoforms in all conditions assayed (Figure 2B). Otherwise, this process can be altered or modulated by the antifungal agent UDA, since its presence affected both the splicing of *T. rubrum Ste20/pakA* mRNA and the relative expression of this gene (Figure 4 and Figure 5). According to the in silico analysis, both STE20/PAKA isoforms appear to be transient molecules, likely with a rapid protein turnover. This hypothesis is based on the presence of the putative PEST domain in both CRIB and catalytic kinase subunits (Figure S1E), since the PEST sequence was postulated to be involved in protein degradation [45]. Moreover, this evidence could support a quick change in cellular response and an even faster change in the signaling pattern. Thus, the data presented here indicate that the retention of intron-1 in *T. rubrum* is modulated by external stressors, such as the presence of antifungal drugs and nutrient availability. The data suggest that in the presence of UDA, fungal cells strongly require the PAKA enzyme without the CRIB domain, probably for a faster response to environmental changes. After 12 h, several drug tolerance genes probably have already been expressed, and the intron-1 retention levels decrease, maintaining a basic level since other stressors, such as the availability of nutrients, also come into effect (Figure 4).

Besides describing a novel biological mechanism of enzymatic activation by AS, these findings suggest that Ste20/PAKA kinase is a crucial molecule in the processes of adaptation and interaction with the host tissue. Moreover, the results suggest that PAKA is a critical virulence factor for *T. rubrum*, as described for *Candida albicans*, which, in its pathogenic form, depends on its STE20 homolog (CST20) for hyphal growth, colonization of the host tissue, and consequent virulence [46,47]. All these pieces of evidence suggest that gene expression and its modifications likely occur for more efficient signaling transduction and, consequently, an appropriate cellular response in a challenging and constantly changing environment. Thus, STE20/PAKA may be a relevant target for the development of novel and specific drugs against dermatophytes. However, a complete understanding of the alternative splicing, as a cellular activation mechanism, requires further experimental substantiation. Heterologous expression and biochemical characterization of both STE20/PAKA kinase isoforms may be fundamental steps to achieve this goal.

## 4. Materials and Methods

### 4.1. Strains and Culture Conditions

*T. rubrum* strain CBS 118892, obtained from the Centraalbureau voor Schimmelcultures in the Netherlands, was cultured as previously described [41]. All assays were carried out in triplicate.

### 4.2. RNA Extraction, cDNA Library Construction, and High-Throughput Sequencing

RNA extraction, cDNA library construction, and high-throughput sequencing were conducted as previously described [41].

### 4.3. Data Analysis

The reads were quality filtered using the software FastQC, aligned with the *T. rubrum* genome available in the Broad Institute’s Dermatophyte Comparative Database (ftp://ftp.broadinstitute.org/pub/annotation/fungi) (accessed on 5 August 2017), using the Bowtie2 algorithm [48], and inspected with the software Integrative Genomics Viewer (IGV) [49]. Furthermore, the independent filter, which precedes the statistical filter, was applied to identify gene-by-gene variances among biological replicates, followed by FDR [50]. These analyses were performed using the DESeq package and manipulated in the R statistical environment [51].

A transcriptome-wide survey of alternative splicing in differentially expressed genes after exposure of *T. rubrum* to UDA was conducted using analysis of IR. This analysis was performed through ad hoc Perl scripts, using only the single-end libraries. Introns were identified in the reference genome and aligned against the sequenced libraries, and read counts were analyzed for each retained intron [41]. qRT-PCR was used to confirm the properties of genes of interest (primers are listed in Table S1). RNA-Seq data were deposited in the Gene Expression Omnibus (GEO) [52] database under accession number GSE102872 (https://www.ncbi.nlm.nih.gov/geo/query/acc.cgi?acc= GSE102872) (accessed on 6 February 2018).

### 4.4. RT-PCR and qRT-PCR Analysis

For gene analysis assays, about 1 × 10^6^ conidia were inoculated into 100 mL of Sabouraud medium and incubated at 28 °C under shaking for 96 h. The resulting mycelia were transferred to Sabouraud medium without drugs (control) or in the presence of UDA (11.25 µg/mL or 25 μg/mL). Then, the mycelia were incubated for 3 h, 12 h, and 24 h at 28 °C, under constant agitation. Gene expression was quantified by qPCR with the StepOnePlus Real-Time PCR system (Applied Biosystems, Foster City, CA, USA). Independent samples from each time point were used, with biological and technical replication performed in triplicate. Each qPCR reaction was performed in a final volume of 12.5 μL, containing 1 μL primer, 6.25 μL SYBR Green PCR Master Mix (Applied Biosystems, Warrington Cheshire, UK), and 50 ng cDNA. The cycling conditions included an initial PCR step of 95 °C for 10 min, followed by 40 cycles of 95 °C for 15 s and 60 °C for 1 min. Expression was assessed based on the relative quantification of responsive genes using the 2^−∆∆*C*t^ method [53]. Data were normalized with two endogenous controls, DNA-dependent RNA polymerase II (rpb II) and DNA topoisomerase II [54]. The reference sample was taken at 0 h. Statistical analysis was performed by one-way analysis of variance (ANOVA) with Tukey’s *post hoc* test using Graph Pad Prism v. 5.1 software. Significance was indicated by *p* < 0.05.

## Supplementary Materials

Supplementary materials can be found at http://www.mdpi.com/1422-0067/19/11/3654/s1. Figure S1: Graphical representation of functional STE20/PAKA protein. Figure S2: Agarose gel electrophoresis (1.5%) run with PCR products from three biological replicates for validation of the pakA/Ste20 intron-1 retention. Table S1: Primers used for qRT-PCR.

## Abbreviations

AMD	Amidation
CRIB	Cdc42/Rac interactive binding
FDR	False discovery rate
GBB	G*β* binding
IR	Intron retention
MAPK	Mitogen-activated protein kinase
MIC	Minimum inhibitory concentration
MYR	N-myristoylation
N-gly	N-glycosylation
PAK	p21 activated kinase
PTMs	Post-translational modifications
RNA-Seq	RNA-Sequencing
RT-PCR	Reverse transcription polymerase chain reaction
qRT-PCR	Quantitative reverse transcription-PCR
UDA	Undecanoic acid
