# Supplementary material for: STE20/PAKA Protein Kinase Gene Releases an Autoinhibitory Domain through Pre-mRNA Alternative Splicing in the Dermatophyte Trichophyton rubrum
Source: Int J Mol Sci. 2018 Nov 20;19(11):3654. doi: 10.3390/ijms19113654 (PMC6274995; doi:10.3390/ijms19113654)
Supplement: Supplementary file 1 [file ijms-19-03654-s001.pdf]

Activation of STE20/PAKA protein kinase in the dermatophyte *Trichophyton rubrum* through alternative splicing

Supplementary Material

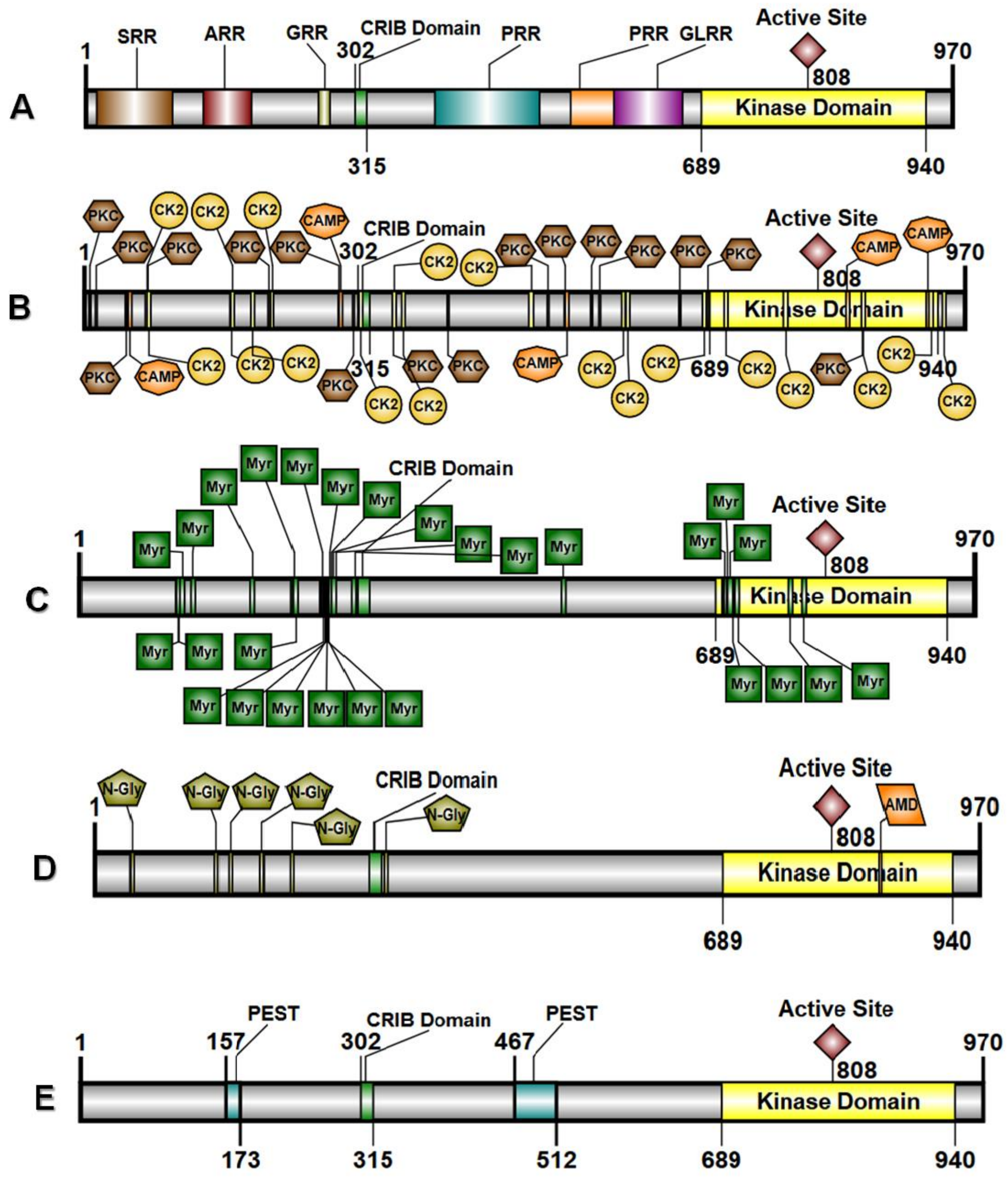

Figure S1: Graphical representation of functional STE20/PAKA protein. **A** - Specific amino acids rich regions; **B** – Phosphorylation sites; **C** – N-myristoylation sites; **D** - N-Glycosylation and amidation sites. **E** – PEST motifs sites; **Legends: SSR:** Serine-rich region; **ARR:** Asparagine-rich region; **GRR:** Glycine-rich region; **PRR:** Proline-rich region; **GLRR:** Glutamine-rich region; **PKC:** Protein kinase C; **CAMP:** cAMP/cGMP-dependent protein kinase; **CK2:** Casein kinase; **Myr:** N-myristoylation; **N-Gly:** N-Glycosylation; **AMD:** Amidation.

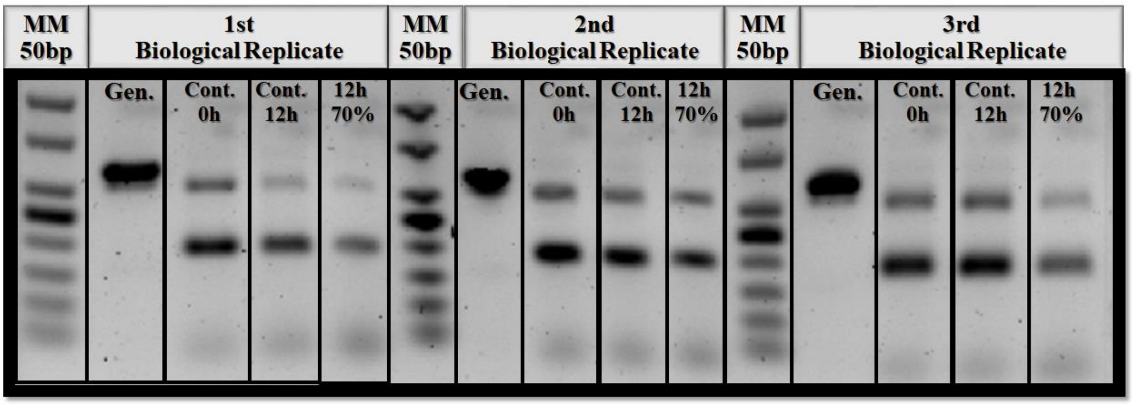

Figure S2: Agarose gel electrophoresis (1.5%) run with PCR products from three biological replicates for validation of the *pakA/Ste20* intron-1 retention. **Legends: Gen.:** Amplification from genomic DNA (gene amplification positive control); **Cont.:** Control condition, culture without the presence of the UDA; **0h:** Amplification product from cDNA coming from the culture in medium without the presence of the drug; **70%:** Amplification product from cDNA coming from culture medium containing 70% of the UDA MIC; **MM:** Molecular weight marker (50bp).

Table S1: Primers used for qRT-PCR.

Primer	Sequence (5′-3′)	Tm	%GC	Lenght (nt)
pakA-F	GCAACTTCGTCAACAGCA	58	50	18
pakA-R	CAACCATTGTCTGCGGAT	60	50	18
i-qRT-pakA.F	CACCACCTTTCTTTCTCC	57	50	18
i-qRT-pakA.R	CAGGATAGTGAAGCAGAC	56	50	18
e-qRT-pakA.F	CCAGAACTACCCTCTACAAC	59	50	20
e-qRT-pakA.R	GGAGGTAGTGGCATTTGT	59	50	18
